# Reno-protection of Urine-derived Stem Cells in A Chronic Kidney Disease Rat Model Induced by Renal Ischemia and Nephrotoxicity

**DOI:** 10.7150/ijbs.37550

**Published:** 2020-01-01

**Authors:** Chao Zhang, Sunil K. George, Rongpei Wu, Parth Udayan Thakker, Mehran Abolbashari, Tae-Hyoung Kim, In Kap Ko, Yuanyuan Zhang, Yinghao Sun, John Jackson, Sang Jin Lee, James J. Yoo, Anthony Atala

**Affiliations:** 1 Wake Forest Institute for Regenerative Medicine, Wake Forest School of Medicine, Medical Center Boulevard, Winston-Salem, NC, USA; 2 Department of Urology, Changhai Hospital, the Second Military Medical University, 168 Changhai Road, Shanghai, People's Republic of China; 3 Department of Urology, First Affiliated Hospital of Sun Yat-Sen University, Guangzhou, Guang Dong, People's Republic of China.; 4 Department of Urology, Wake Forest Baptist Medical Center, Medical Center Boulevard, Winston-Salem, NC, USA; 5 Department of Urology, College of Medicine, Chung-Ang University, Seoul, South Korea

**Keywords:** Urine-derived Stem Cell, Chronic Kidney Disease, Kidney degeneration

## Abstract

**Purpose:** Drug-induced nephrotoxicity can occur in patients with pre-existing renal dysfunction or renal ischemia, potentially leading to chronic kidney disease (CKD) and end-stage renal disease (ESRD). Prompt treatment of CKD and the related side effects is critical in preventing progression to ESRD. The goal of this study was to demonstrate the therapeutic potential of urine-derived stem cells (USC) to treat chronic kidney disease-induced by nephrotoxic drugs and renal ischemia.

**Materials and methods:** Human USC were collected, expanded and characterized by flow cytometry. A CKD model was induced by creating an ischemia-reperfusion injury and gentamicin administration. Twenty-eight adult immunodeficient rats were divided into three groups: PBS-treated group (n=9), USC-treated group (n=9), and sham group with age-matched control animals (n=10). Cell suspension of USC (5 x 10^6^ / 100µl / kidney) or PBS was injected bilaterally into the renal parenchyma 9 weeks after CKD model creation. Renal function was evaluated by collection blood and urine samples to measure serum creatinine and glomerulus filtration rate. The kidneys were harvested 12 weeks after cell injection. Histologically, the extent of glomerulosclerosis and tubular atrophy, the amount of collagen deposition, interstitial fibrosis, inflammatory monocyte infiltration, and expression of transforming growth factor beta 1 (TGF-ß1), and superoxide dismutase 1 (SOD-1) were examined.

**Results:** USC expressed renal parietal epithelial cells (CD24, CD29 and CD44). Renal function, measured by GFR and serum Cr in USC-treated group were significantly improved compared to PBS-treated animals (p<0.05). The degree of glomerular sclerosis and atrophic renal tubules, the amount of fibrosis, and monocyte infiltration significantly decreased in USC-treated group compared to the PBS group (p<0.05). The level of TGF-ß1 expression in renal tissues was also significantly lower in the PBS group, while the level of SOD-1 expression was significantly elevated in the USC group, compared to PBS group (p<0.05).

**Conclusions:** The present study demonstrates the nephron-protective effect of USC on renal function via anti-inflammatory, anti-oxidative stress, and anti-fibrotic activity in a dual-injury CKD rat model. This provides an alternative treatment for CKD in certain clinical situations, such as instances where CKD is due to drug-induced nephrotoxicity and renal ischemia.

## Introduction

Chronic kidney disease (CKD) is a major health problem characterized by a gradual loss of kidney function over time. Over 30 million people or 15% of US adults are estimated to have CKD 1. CKD may progress to end-stage renal disease (ESRD), which is a public health concern and big economic burden. ESRD costs the United States taxpayers approximately 32.8 billion dollars in annual Medicare expenditures [2]. Causes of CKD vary widely, however drug-induced nephrotoxicity has been an increasingly recognized complication of many therapeutic agents in the clinical setting. In patients with pre-existing renal dysfunction or renal ischemia, the effects of drug-induced nephrotoxicity can be profound and may accelerate progression to ESRD 2. At present, hemodialysis and renal transplantation are both effective treatment modalities for ESRD. However, hemodialysis is expensive with many potential complications while renal transplant as a treatment modality is limited by the number organ donors available^3^ .

Cell therapy has emerged as a promising therapeutic approach for the treatment of CKD with great potential 4. Several stem cell types from a variety of sources have been used in animal experiments 5-10. Bone marrow-derived stem cells were found to significantly improve renal function in mice models 5, and their safety was subsequently studied in a phase 1 clinical trial 11. In addition, stem cells derived from amniotic fluid 6,7, endothelium 8, adipose tissue 9 and primary renal cells 10 have demonstrated therapeutic effect in renal regeneration. However, obtaining these stem cells requires invasive procedures, which may cause iatrogenic injury to adjacent organs and increases infection risk among other complications. Thus, the desire to obtain stem cells from a simple, non-invasive and inexpensive route is of utmost necessity.

Recently, we demonstrated that stem cells exist in human urine and possess the potential for clinical applications 12-20. Urine derived stem cells (USC) are thought to originate from the parietal cells in renal glomeruli 14 and have the potential for tissue regenerative effects, including robust proliferative potential, multi-potential differentiation, and the ability to exert regenerative effects via paracrine factors 14,21,22. USC secrete multiple paracrine factors to recruit native cells to participate in tissue regeneration and induce immune-modulatory changes *in vivo*. Studies from our group and other laboratories have demonstrated that USC or the media derived from USC significantly improved renal function by reducing inflammation and fibrin matrix deposition within the kidney in different rodent CKD models, including age-related kidney disease 23 and streptozotocin-induced diabetic nephropathy 24,25. Furthermore, USC enhanced renal function in an acute renal ischemia model 26. Nephrotoxic drug induced renal damage superimposed on pre-existing renal damage is one of the more common causes of iatrogenic CKD 2. However, it is challenge to revise the renal function and prevent histological structure damages from the chronic renal damages in clinical settings. Compared to stem cells derived from other sources such as amniotic fluid, placenta, bone marrow, adipose tissues, USC have unique advantages in the treatment of CKD. USC can be obtained from simple, safe, non-invasive and low-cost approaches. A large of amount of stem cells can be generated through a few weeks. In addition, as the kidney tissue specific stem cells, USC might be optimal for the kidney tissue repair. As such, the goal of this study was to determine therapeutic impact of USC on renal parenchyma architecture and renal function in a rat CKD model, induced by dual nephrotoxic drug-renal ischemia injury.

## Methods

### USC isolation and identification

Collection of human urine samples for USC isolation in this study was approved by the Wake Forest University Health Sciences Institutional Review Board. USC culture and characterization have been described previously 12-20. In brief, USC were collected from healthy adult males (n=6) and expanded in culture media comprised of keratinocyte serum-free medium (Gibco, Gaithersburg, MD) and Dulbecco's Modified Eagle's medium (DMEM, high glucose, Gibco, Gaithersburg, MD) with 5% fetal bovine serum (FBS) (Gibco, Gaithersburg, MD) containing supplements 12, see **Table 1**. Expanded USC were trypsinized and incubated with anti- human antibody labeled with cell surface markers of renal parietal epithelial cells (CD24, CD29 and CD44) (BD, Franklin Lakes, NJ) 27, mesenchymal stem cells (MSC) (CD73, CD90, CD105, and CD146) (BD, Franklin Lakes, NJ), embryonic stem cells (SSEA4), hematopoietic stem cell (CD31, CD34, CD45) (BD, Franklin Lakes, NJ) and STRO-1 (BioLegend, San Diego, CA). Fluorescein isothiocyanate (FITC) or phycoerythrin (PE) conjugated isotype antibodies were used to determine background fluorescence. All cells were analyzed using BD FACS Caliber analytical fluorescence activated cell sorter (BD, Franklin Lakes, NJ).

### Osteogenic and adipogenic differentiation of USC

#### Adipogenic induced differentiation

USC were seeded at a density of 21,000 cells/cm^2^ and cultured in serum containing DMEM low-glucose medium with 1 µM dexamethasone, 500 µM 3-isobutyl-1-methylxanthine, 10 µg/ml insulin, and 100µM indomethacin for 28 days with medium changes every third day. Differentiated cells were then fixed with 4% paraformaldehyde for 30 min at room temperature and stained with fresh 0.3% Oil Red O solution for 50 min. An inverted microscope was used to identify red stained areas, indicating fat droplets. Adipose derived stem cells were used as positive controls.

#### Osteogenic induced differentiation

USC were seeded at a density of 4,000 cells/cm^2^ and cultured in serum containing DMEM low-glucose medium with 100 nM dexamethasone, 10 mM β-glycerophosphate and 50 µM ascorbic acid-2-phosphate (Wako Chemicals, Richmond, VA) for 28 days with medium changes every third day. Differentiated osteocytes were then fixed by ice-cold 95% ethanol for 5 minutes at 4 ℃ and stained for calcium deposits with 2% Alizarin Red Solution (pH 4.0). An inverted microscope was used to identify orange-red stained areas, indicating calcium deposits.

### An athymic rat model of dual injury-induced CKD

Animal experiments were approved by Wake Forest Institutional Animal Care and Use Committee (Protocol no A11-085). Animals were housed in a temperature-controlled environment with free access to food and water. A 12-hour light and 12-hour dark cycle was provided.

To avoid the potential risk of immunoreaction caused by the implanted xenogenic cells, athymic rats (RNU316) were used for this study. These athymic nude rats were T-cell deficient and demonstrated depleted cell populations in thymus-dependent areas of peripheral lymphoid organs. In total, 58 male athymic rats (age 9 weeks, Charles River, NC) were used in this study. To optimize the doses of gentamicin and renal ischemia time frame, 30 animals were used for creation model of dual injury-induced CKD before USC or PBS injection. Among these animals, 22 animals died and 8 animals recovered renal function (failure of model creation) during the model creation processes.

**Figure 1:**
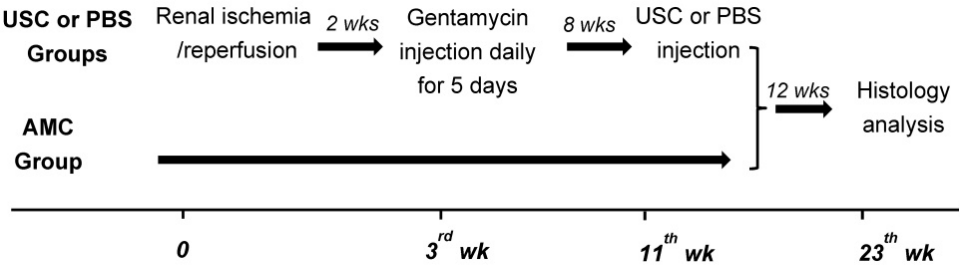
** A brief flow chart of the study design.** The CKD model was created using dual-injury of renal ischemia and nephrotoxicity using 5 consecutive days of gentamycin injection, 2 weeks after renal ischemia-reperfusion injury. Eight weeks after confirmation of model creation using serial serum Cr and GFR, animals received either USC or PBS via direct injection into renal parenchyma. Renal function was performed at different time points. Finally, histological and immunocytochemical analysis were performed 12 weeks after injection. Age matched animals were applied as controls (AMC).

Once the model was successfully established, 28 animals were divided into three groups: a PBS-treated group (n=9), an USC-treated group (n=9), and an age-matched control group (n=10). A CKD model was induced by creating an ischemia-reperfusion injury and gentamicin injection as demonstrated in **Figure 1** 28. Briefly, the rats were anesthetized and laid in the supine position with continuous 4% isoflurane via nose cone. A midline incision was made and intestines were retracted to expose both kidneys. Using vascular clamps, the renal pedicles were clamped bilaterally, for 60 minutes and then released to induce ischemia-reperfusion injury. The clamps were then removed and the incision was closed. Two weeks after surgically-created renal ischemia injury, gentamicin (100 mg/kg/day, Phoenix Pharmaceutical Saint Joseph, MO) was injected subcutaneously for 5 consecutive days. Serum creatinine levels were used to evaluate renal function. Blood and urine samples were collected every 2 weeks and analyzed with blood chemistry machine (Beckman Coulter Inc., Brea, CA). Glomerulus filtration rate (GFR) was calculated as follows: GFR=urine creatinine x urine volume/serum creatinine/time.

### Injections of USC into rat renal parenchyma

Eight weeks after the dual injury model was created, a USC cell suspension was injected into the kidneys for the USC-treated group. The abdominal contents were exposed once again and both kidneys were identified. A cell suspension of USC was administered into the parenchyma of both kidneys. Each kidney received a total of 5x10^6^/ml in 100 µl of PBS, 8 weeks after CKD creation. To distribute injected cells evenly, 50% of the aforementioned cell suspension was injected into the upper pole and lower pole of each kidney. PBS (100 µl) only was injected in the same fashion as above for the PBS-control group. The model was not created in aged-matched control animals and no injections were performed. Blood and urine samples were collected to test renal function parameters i.e. serum creatinine and GFR, at 2 week intervals. Kidneys were harvested from each animal for histological evaluation, 12 weeks after injection.

### Histological and Immuno-histochemical analysis

Harvested kidney tissues were fixed in 10% paraformaldehyde solution and processed for paraffin embedding. Five µm sections were obtained. Slides were stained with hematoxylin and eosin (H&E), and numbers of glomeruli were counted under high power fields (200X) microscope (Leica, Germany). Normal glomeruli are uniform in size, visceral (inner) structures and Bowman's space. Amounts of collagen deposited were assayed by computerized ImageJ (ImageJ software package, NIH) based on Masson's Trichrome staining. Immunohistochemistry was performed with anti-human leukocyte antigen (HLA) and anti CD68 antibodies as a marker of macrophage (BD Biosciences, CA). All slides were blocked in serum-free protein blocker (DAKO, Carpinteria, CA) for 20 minutes.

Transforming growth factor beta 1 (TGF-ß1, DAKO) was tested as dysregulation of TGF-β activation and signaling may result in apoptosis of resident functional cells but proliferation of fibroblast. In addition, superoxide dismutase 1 (SOD-1) as an antioxidant enzyme protecting the cell from reactive oxygen species toxicity were tested in USC-treated group, compared to PBS-treated control. Anti-superoxide dismutase 1 antibody (EPI722Y) was used at a 1:400 dilution. After incubation with the primary antibodies, tissue sections were incubated with biotin-conjugated secondary antibodies (1:300 dilution) (Vector Laboratories, Burlingame, CA, USA) at room temperature for 60 min, followed by incubation with HRP-conjugated streptavidin (Vector Laboratories) for 30 min at room temperature and further developed with a DAB substrate kit (Vector Laboratories).

Positive cells were counted under lower and high power fields (100X, 400X). Total number of glomeruli with abnormal structures, increased glomerular size, pathological changes of renal tube, intestinal fibrosis, collagen deposits and inflammatory were quantified. The degree of histological abnormalities on nephrons, such as dilation or collapse, epithelium and obliterated lumen necrosis in renal tubule and glomerular was evaluated. Quantitative comparison of histology and immunohistochemical staining was measured by digital image analysis.

### Statistical analysis

All data were analyzed using software Statistical Package for the Social Sciences (SPSS) version 16.0 for windows (SPSS, Inc., Chicago, IL). Normality of the analyzed data was corroborated with Shapiro-Wilk test. When parametric analysis was possible, data were expressed as mean ± SD and one-way analysis of variance (ANOVA) was used for comparison among the three groups. Least Significant Difference test was applied to further compare differences among three groups. P<0.05 was considered statistically significant.

## Results

**Figure 2:**
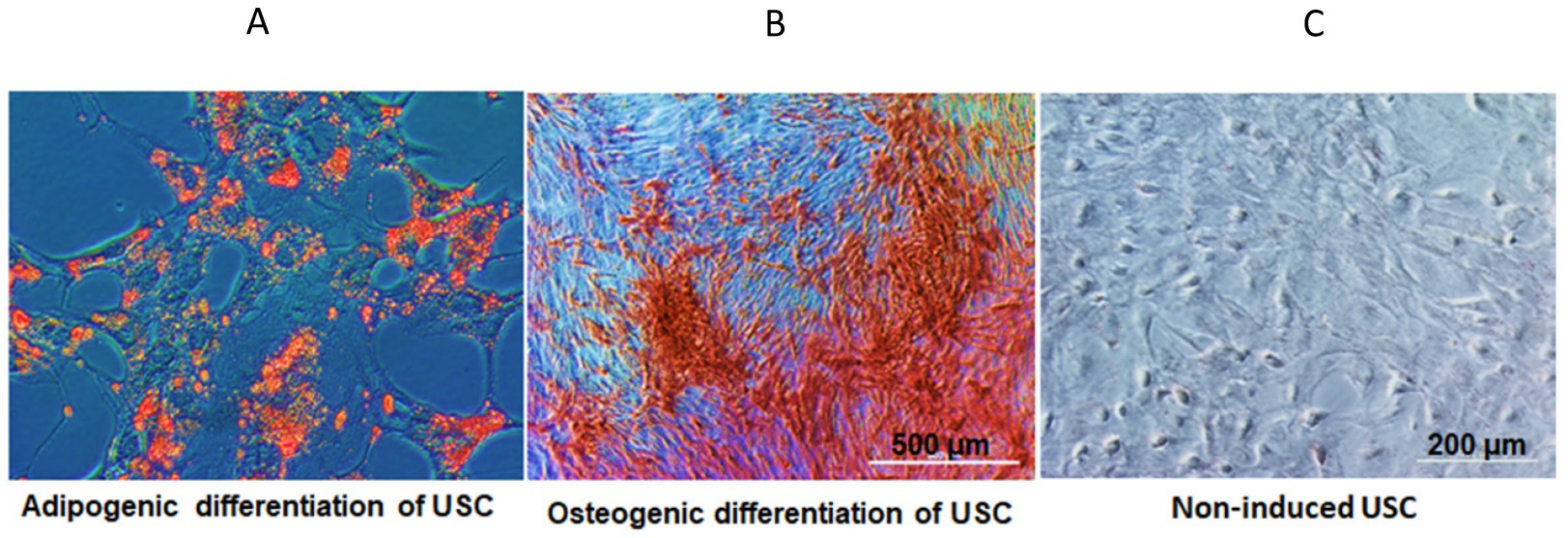
** In vitro differentiation potential of USC.** (**A**) Adiopogenic differentiation of USC was demonstrated with oil red O (lipid droplet). (**B**) Osteogenic differentiation of USC was demonstrated with Alizarin Red. (**C**) Non-induced USC acted as a control group.

Individual USC appeared consistently as rice grain-like at primary culture (**Supplementary Figure 1**). USC at passage 3-5 were strong positive for glomerular parietal epithelial cells (CD24, CD29 and CD44), and also classical cell surface markers for MSC (CD73, CD90, CD105, CD146 and SSEA-4), but lack of the expression of hematopoietic markers (CD 31, CD34 and CD45). These results were consistent with our previous reports (**Supplementary Figure 1**) 12-20. In addition, USC differentiated into osteogenic and adipogenic cell lineages, respectively (**Figure 2**).

**Figure 3:**
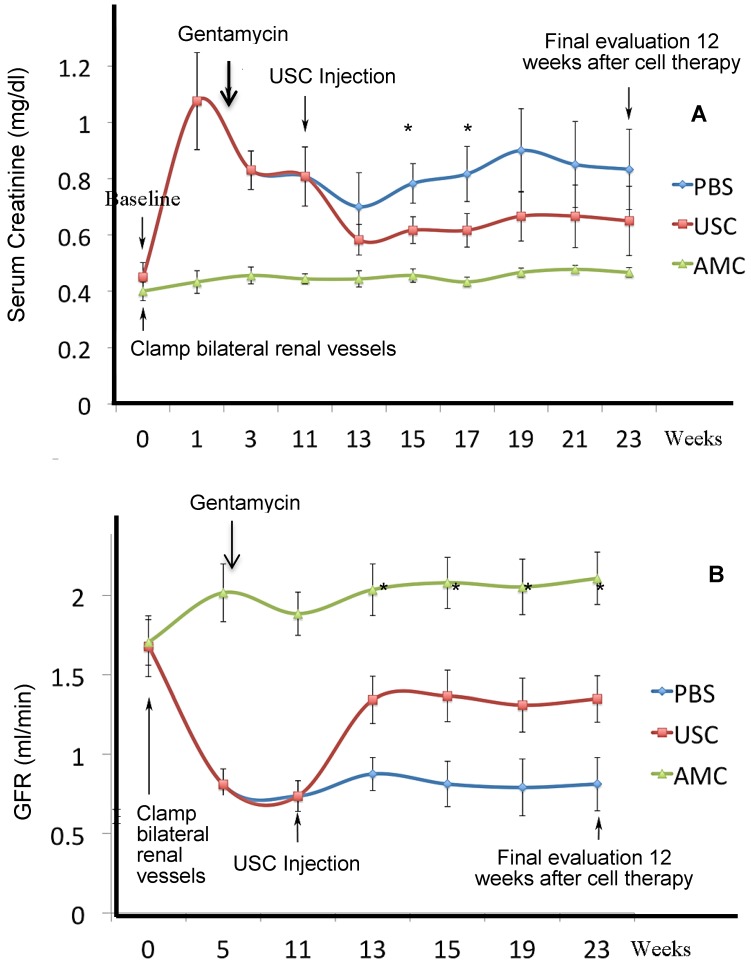
** Dynamic changes of renal function at different time points**. A CKD model was successfully established in athymic rats. (**A**) Serum creatinine levels were significantly lower in USC-treated rats, compared to PBS-treated group on weeks 4 and 6 after cell therapy, respectively. (**B**) Glomerular filtration rate was significantly higher in USC-treated group at each time period after cell therapy, compared to the PBS-treated group (* p<0.01).

For evaluation of renal function, serum creatinine (Cr) was evaluated and GFR was calculated every 2 weeks. Creatinine rise and GFR depression were used as indicators of effective CKD model creation. Creatinine levels increased significantly in all animals assigned to USC and PBS groups after one week, post-model creation and remained elevated compared to AMC (p<0.01). Likewise, GFR declined significantly for animals assigned to USC and PBS groups after one week, post-model creation (p<0.01). The changes in creatinine and GFR were similar in both USC and PBS groups until injection was performed at 13 weeks post-model creation. In the USC-treated group, serum creatinine decreased and GFR increased significantly improved compared to PBS-treated animals two weeks after cell implantation (p=0.027 and p=0.037, respectively). Th difference in GFR and Cr remained significantly different between these groups until the study endpoint 12 weeks after cell implantation. GFR in the USC-treated group improved to 50% of that in AMC whereas PBS-treated animals only exhibited a 25% recovery of GFR compared to AMC (**Figure 3**).

**Figure 4:**
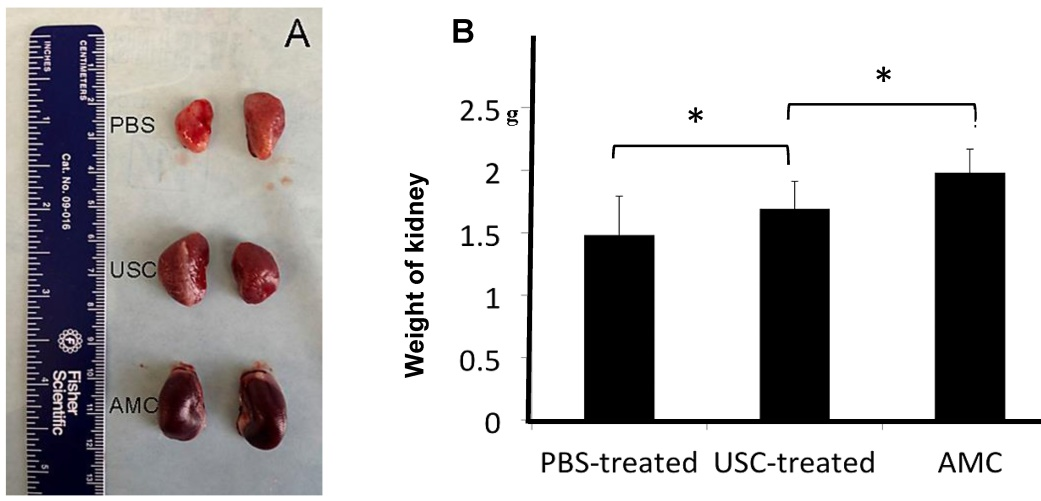
** The gross appearance and weight changes of the kidneys.** (**A**) The gross appearance of kidneys in the three treatment groups was noted. (**B**) The weight of the kidneys in USC-treated rats was significantly elevated compared to the PBS-treated animals but also significantly lighter than that in AMC group, * Significance at the *p<0.05* level.

Grossly, the surface of the kidneys in PBS-treated group was coarse and pale, while kidneys in USC-treated group appeared similar to AMC (**Figure 4**). In addition, the kidney weight in PBS-treated group (1.48±0.31g) was significantly lower compared to those of the AMC (1.98±0.19 g) and USC-treated groups (1.69±0.22 g) 12 weeks after injection therapy. The kidney weight in USC-treated group significantly increased compared to that in PBS-treated group (p<0.01) despite not returning to kidney weights exhibited in the AMC group.

**Figure 5:**
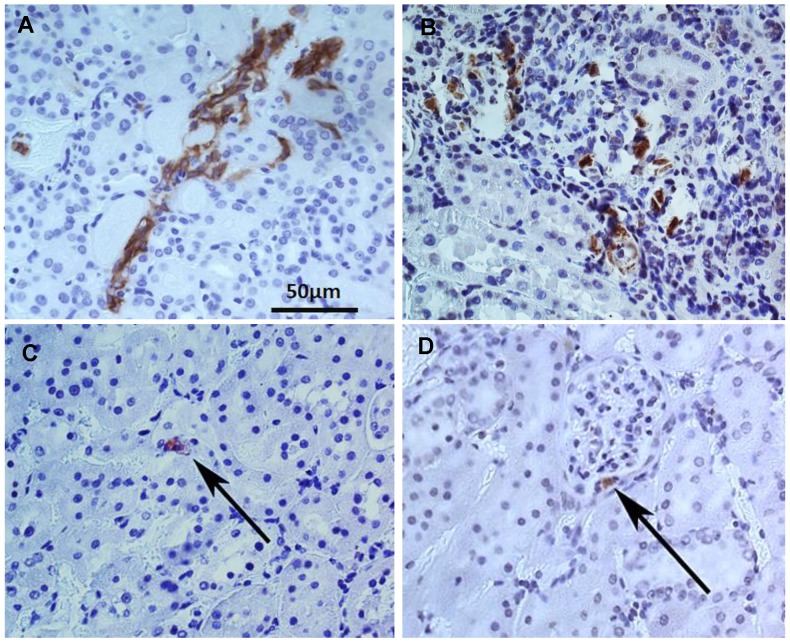
** Tracking implanted USC within renal tissues by immuncytochemistry analysis with anti-HLA A antibody**. More grafted USC (brown) were found in the tubule interstitial regions one week (**A**) and two weeks (**B**) after implantation compared to 12 weeks. Number of the implanted USC decrease with time during the 12 week follow-up. A few cells were still identified in the renal tubules and the medulla (**C**) as well as around Bowman's capsule in the renal cortex (**D**).

**Figure 6:**
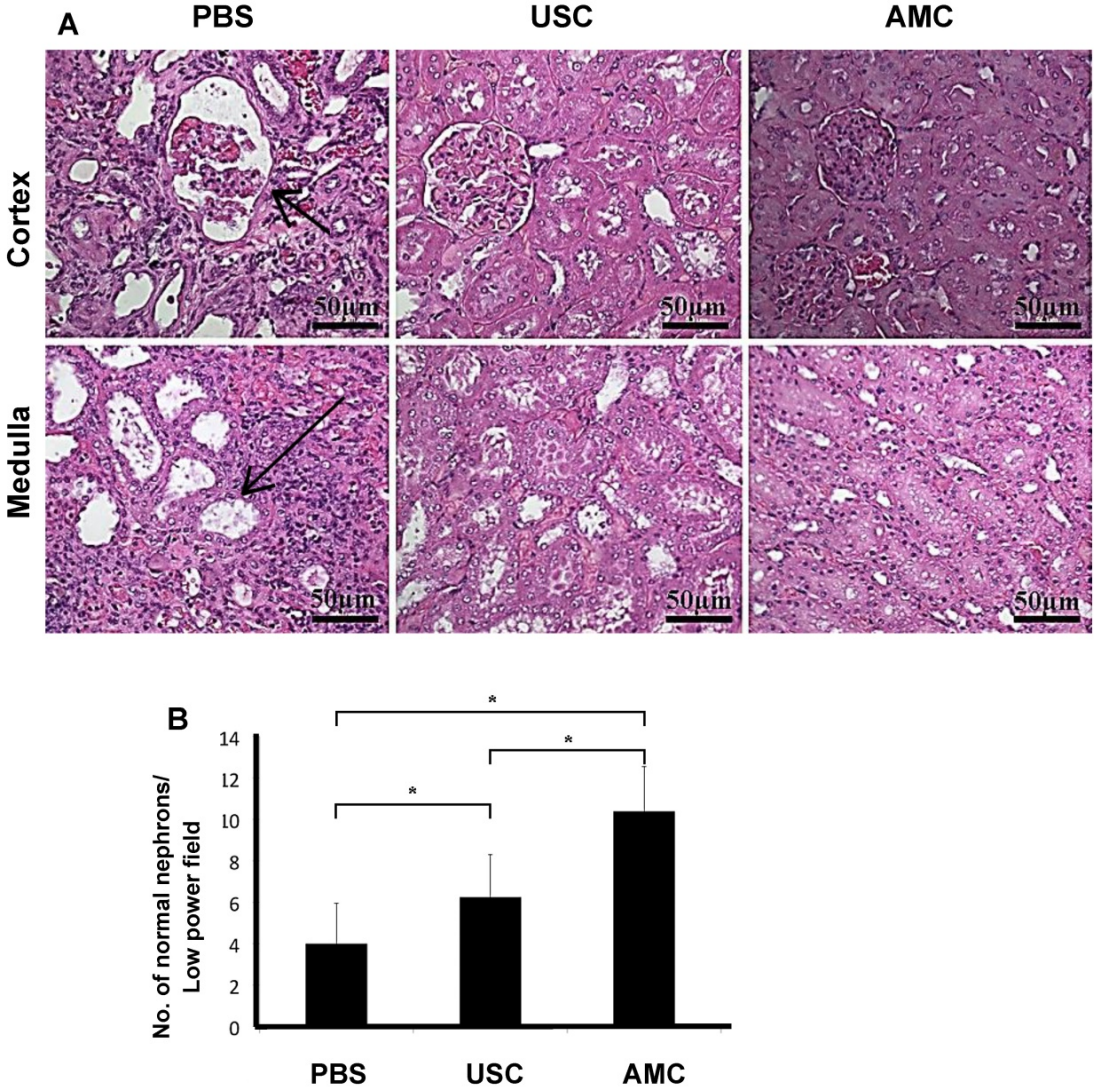
** of Patho-histological changes in nephrons within renal cortex and medulla after USC implantation. (A)** About 60% of glomeruli increased in size with collapse of some glomerular tufts (short arrow) within the renal cortex and 62% of renal tubules were dilated (long arrow) within the medulla in PBC-treated rats. In contrast, a majority of glomeruli and renal tubules displayed normal structure and only 40% of nephrons displayed abnormal configuration in USC-treated group. (**B**) Expressed as the average numbers of relative abnormal nephrons in six fields per sample at 200x magnification. Numbers of normal nephron within renal tissue under high power field with H&E staining. * Significance at the *p<0.05* level.

Immunocytochemical analysis using HLA staining revealed that the implanted human USC were present around Bowman's capsule or scattered within renal tubules for the entire study period, however numbers of the grafted cell decreased significantly, 12 weeks after injection compared to immediately post-injection (**Figure 5**). To determine the reno-protective effects of USC, we evaluated the changes in glomerular, renal tubular, and tubulo-interstitial structure among the three groups. In the PBS-treated group, about 38% of glomeruli displayed normal architecture (4.0±2.0 normal glomeruli/high power field [HPF]) within the renal cortex while 62% (6.6±2.0) demonstrated glomerulosclerosis evidenced by wrinkling and collapse of the basement membrane and constricted glomerular capillaries, compared to AMC. This pathological changes in nephrons were significantly lower than the USC-treated group (6.3±2.1) and the AMC group (10.6±2.2) (p<0.01). Conversely, the USC-treated group demonstrated 60% normal glomeruli with 40% displaying glomerular sclerosis and dilated, atrophic tubules (**Figure 6**) compared to the AMC group (p<0.01).

**Figure 7:**
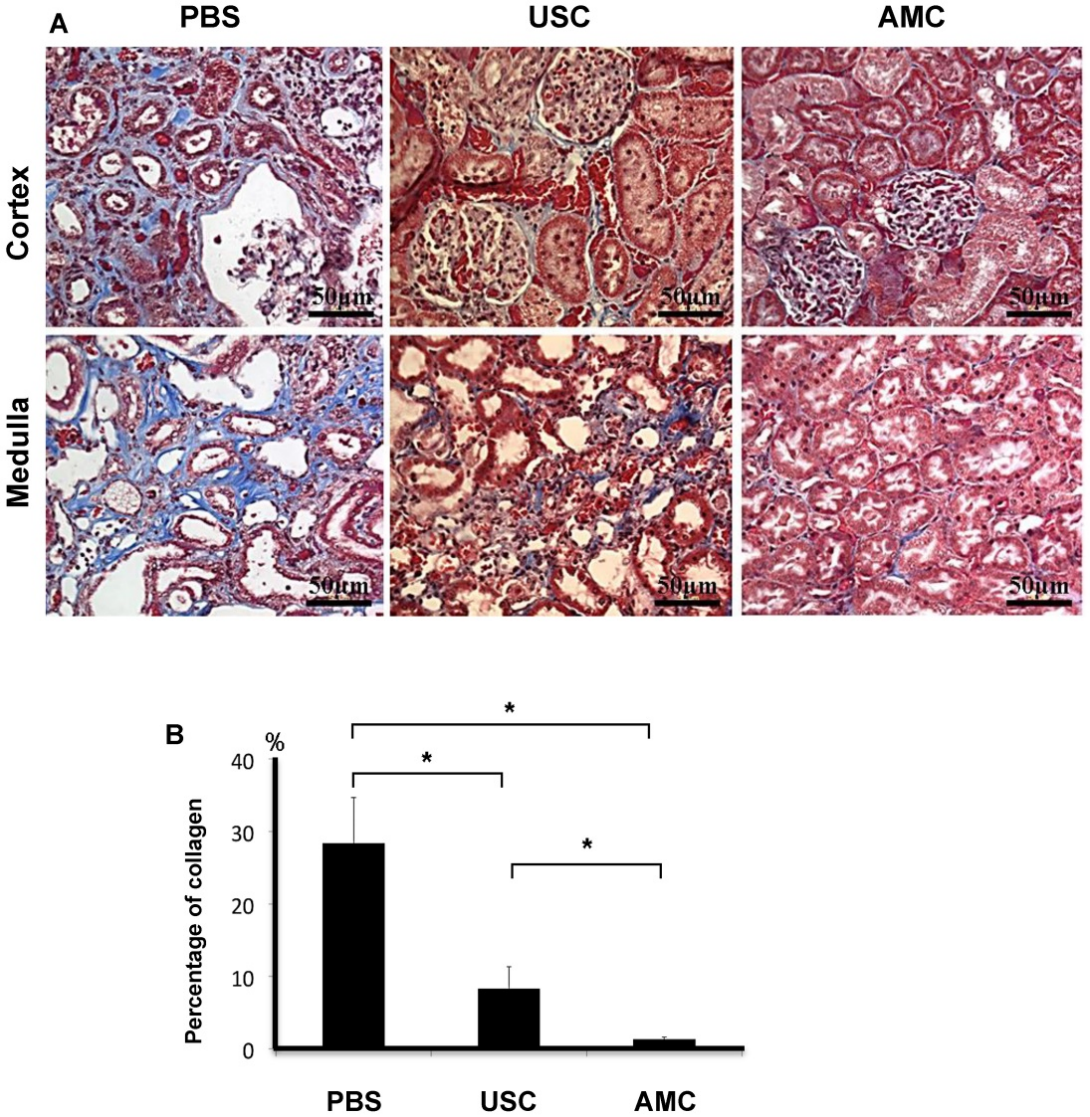
** Histological appearance of collagen deposition and fibrotic changes of the kidneys**. (**A**) Among of collagen deposition in Masson Trichrome staining was significantly elevated in PBS-treated rats, and minimal changes in kidney of rats treated with USC-treated group compared to that in AMC. (**B**) Percentage of collagen deposition within the renal parenchyma. * Significance at the *p<0.05* level

**Figure 8:**
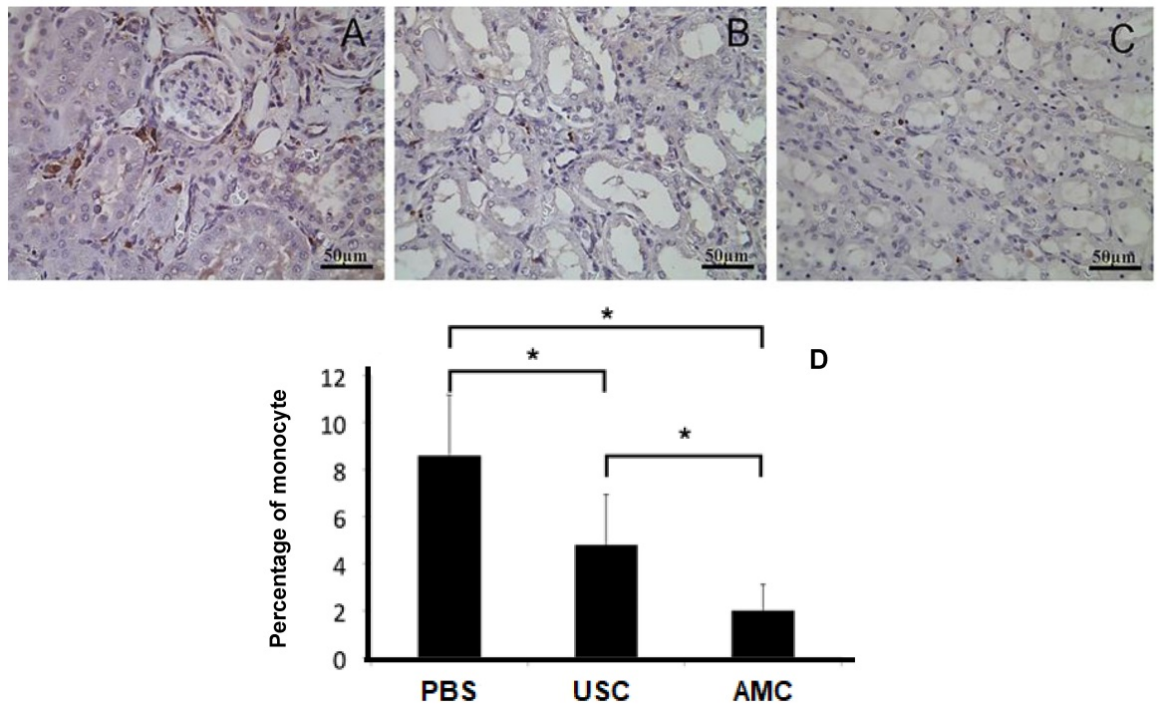
** CD68 expression as a surrogate for monocyte infiltration within the kidney**. (**A**) Number of infiltrated monocyte expressing positive for CD68 significantly increased in PBS-treated group, compared to AMC. (**B**) Number of cells expressing CD68 significantly decreased in USC group compared (**C**) compared to PBS group. (**D**) Analysis of CD68 expression in the three groups with immunocytochemical staining. * Significance at the *p<0.05* level

Collagen deposition in the renal parenchyma was investigated using Masson's Trichrome staining (**Figure 7**). Collagen deposition was elevated in PBS-treated animals (28.4±6.3). This was significantly higher than in USC-treated group (8.3±3.0) and the AMC group (1.3±0.3) (p<0.01), which represented over a 20-fold increase. There was also significantly higher collagen deposition in the USC-treated group compared to the AMC group which was only a 6-fold increase. To assess monocyte infiltration (**Figure 8**), numbers of cells expressing CD68^+^ was significantly higher in the PBS group (8.7±2.54) compared to both USC-treated group (4.7±2.12) and AMC group (2.0±1.05) (p<0.01). The USC-treated group exhibited an elevated numbers of cells expressing CD68^+^compared to the AMC group as well, however albeit not as drastic.

**Figure 9:**
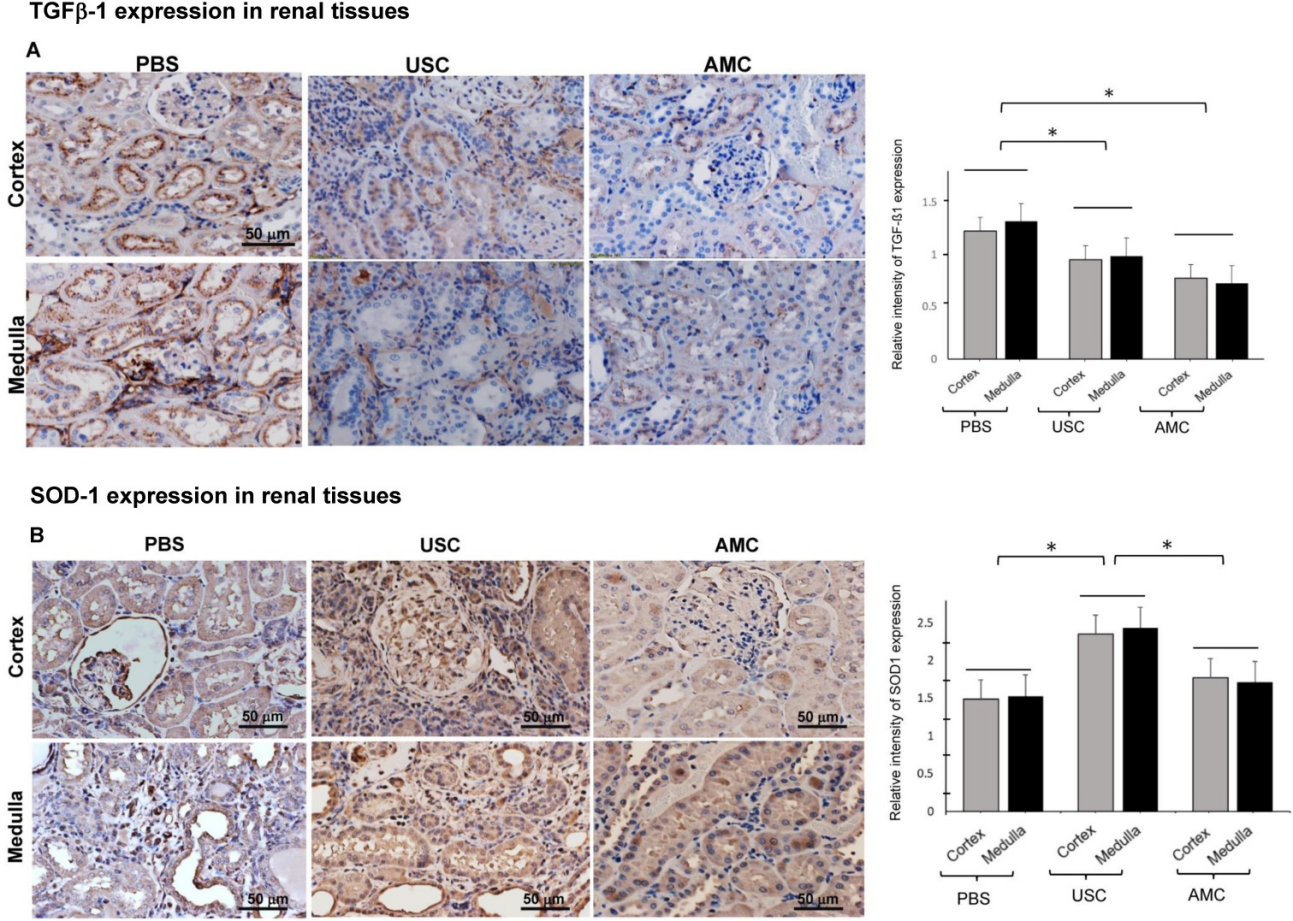
** Effect of USC implantation on anti-fibroblast and redox properties in CKD rodent model.** (**A**) Amount of TGF-β1 expression significantly increased in renal cortex and medullar tissue of PBS treated animals compared to that of AMC group. However, USC implantation significantly decreased levels of TGF-β1 expression, compared PBS treatment. (**B**) Amount of SOD-1 expression levels significantly increased in renal tissue of USC-treated groups compared to that of PBS group. Semi-quantification is expressed as the average relative inte nsity of six fields around defect site per sample, each taken at a 200x magnification. Representative images are shown at 200x magnification. *p < 0.01 compared to AMC as a unit set.

To evaluate tissue fibrosis within the renal tissues, TGF-ß1 as an inflammatory marker (**Figure 9**) was used. TGF-ß1 expression was significantly high in the PBS-treated groups in both the cortex and medulla (1.2±0.1 and 1.3±0.08) which represented a 58% and 69% increase compared to AMC (0.8±0.05 and 0.7±0.06). Expression of TGF- ß1 in the cortex and medulla of the USC-treated group (0.95±0.09 and 0.99±0.06), respectively, represented as 23% and 29% increase compared to AMC. The relative increase in TGF-ß1 for PBS-treated animals was significantly higher than for the USC-treated group (p<0.01).

Superoxide dismutase (SOD-1) as important mediator in the oxidative stress response was assessed (**Figure 9**). SOD-1 expression in the PBS-treated group in the cortex and medulla was 1.49±0.17 and 1.53±0.14, which represented 84% and 89% of that in the AMC group (1.8±0.16 and 1.7±0.11). Expression of SOD-1 in the USC-treated group was 2.35±0.1 and 2.43±0.11 in the cortex and medulla, respectively. This represented a 33% and 42% increase in SOD-1 expression compared to AMC. The relative changes in SOD-1 expression were significantly different among the three study groups (p<0.01).

**Figure 10:**
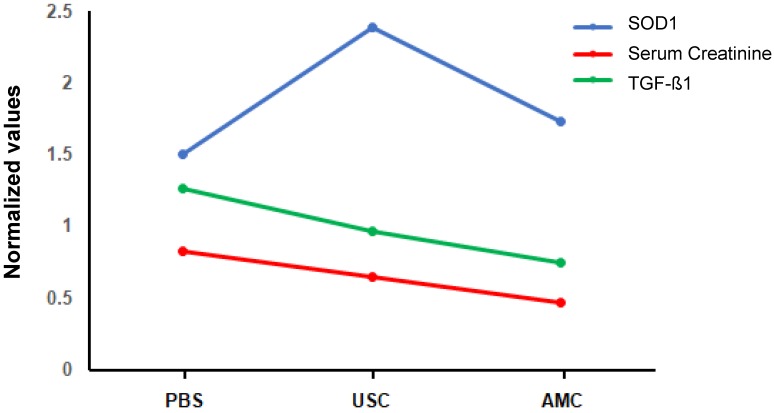
** Relationship between serum creatinine and both the oxidative stress parameter and the inflammatory parameters with regression line analysis.** Antioxidant and antiinflammatory activity of USC is behind the renoprotective activity, compared to those of PBS-treated and AMC groups.

Taken together, there is a tendency between the creatinine clearance and both the inflammatory and antioxidant parameter amount three groups, i.e. renal function improved with less inflammatory and increase level of antioxidate in USC treated group than those in PBS-treated group, competed to AMC (**Figure 10**) .

## Discussion

Chronic kidney disease is caused by progressive injury to nephrons including glomeruli and renal tubules, interstitium and vasculature. Etiologies include genetic predisposition, metabolic derangements, renal ischemia, environmental and infectious causes, autoimmune conditions, and drug-related nephrotoxicity. Repeated or superimposed insults may ultimately lead to chronic kidney disease, which ultimately may progress to end-stage renal disease without appropriate therapy. Of particular interest to us was drug-induced nephrotoxicity.

Drug-induced nephrotoxicity occurs more frequently in patients with pre-existing renal dysfunction or ischemia due to acute kidney injury, chronic kidney disorder, diabetic nephropathy, congestive heart failure, among other causes. Gentamicin is notorious for its nephrotoxic side-effects, which is characterized by proximal convoluted tubular necrosis and glomerular congestion, resulting in decreased GFR and renal dysfunction 29. Similarly, renal ischemia often causes acute tubular necrosis 30. Dual insults with renal ischemia and drug nephrotoxicity may lead to severe CKD and ESRD without prompt treatment and thus it is necessary to develop an effective approach to prevent progression of CKD to ESRD.

Several animal models of CKD have been reported in the literature 31, however, due to the robust regenerative capacity of rodents, it is challenging to study long-term efficacy of cellular therapy. The subtotal nephrectomy model 32-34 requires a complex procedure and does not reflect the mechanism of renal failure most often observed in humans. The streptozotocin model mimics tissue injury observed in diabetic nephropathy. However, as CKD caused by drug-induced nephrotoxicity increases, the need for another animal model is required. Herein, we have established a model using ischemia-reperfusion injury combined with gentamicin-induced nephrotoxicity to provide an easy and reliable animal model to study CKD 28.

Given that definitive treatment i.e. renal transplant is not commonplace; it is of utmost importance to prevent progression of CKD to ESRD. The nephron is the functional unit of the kidney and thus we quantified the number of normal nephrons in each treatment group. In the USC treated group, 70% of glomeruli displayed normal architecture while this number decreased to 40% in the PBS-treated group. In contrast, numbers of abnormal nephrons significantly lower in USC-treated group than those in PBS group. Likewise there was a significant decrease in serum Cr and increase in GFR in the USC group compared to PBS treated animals. It is likely that the preservation of glomerular architecture, evidenced by a lower level of glomerulosclerosis and dilatated, atrophic tubules, in the USC treated group compared to the PBS group is responsible for the chemical (serum Cr) and functional (GFR) findings.

Tissue fibrosis and collagen deposition are important contributors to progressive renal failure. This is a complex process involving fibroblasts, immune reactions and free radical damage to the renal parenchyma. We evaluated these processes through gross examination of the kidneys and through the chemical investigation of mediators of fibrosis and free radical damage. Monocyte infiltration as cells expressing CD68^+^ was elevated in the PBS group to a greater extent than in USC treated group, compared to AMC. TGF- ß1 as a surrogate for tissue fibrosis followed a similar trend. Superoxide dismutase is an important mediator in inhibiting free radical induced tissue damage and its expression was lowest in the PBS treated group and highest in the USC treated group. Given the lower levels of TGF- ß1 and CD68^+^ cells in the USC group compared to the PBS group, it is unsurprising the see a lower level of collagen deposition and improved renal function in the latter (**Figure 9**). Furthermore, collagen and fibrosis lead to glomerular and tubular atrophy, explaining the gross appearance and the weights of the kidneys in the PBS group compared to the USC and AMC groups.

Our study is not without limitations which may need to be addressed prior to beginning safety trials. First, we only administered a single dose of a given concentration of USC. It is feasible that multiple injections may further improve the renoprotection imbued by USC. These injections may need to be distributed over several weeks to months for maximal effect. Second, it is possible that increasing the number of injected cells will improve kidney function even further.

Third, very few studies have been conducted investigating routes of administration for urologic stem cell therapy and thus it may be that intravenous therapy may provide improved benefit over intrarenal injection. Finally, molecular analysis of pro-regenerative, anti-apoptotic or anti-fibrotic pathways needs further investigation.

Herein, we have demonstrated that local implantation of human USC significantly improved renal function, imbued a protective effect on nephrons, reduced renal scaring, and mollified the inflammatory response in a renal ischemia- reperfusion, gentamicin, dual injury-induced CKD rodent model (**Figure 10**). Through our chemical and functional analysis, it is clear that USC have some impact on the aforementioned parameters insofar as USC treatment ameliorates renal degradation seen in the group that received no treatment.

## Supplementary Material

Supplementary figures and tables.

## Figures and Tables

**Table 1:** Antibodies, mediums, and serums used in this study**.**

Items	Antibody	Type	Source	Catalog No.
**List of Antibodies**	CD24	FITC-conjugate	BD	560992
	CD29	PE-conjugated	BD	555443
	CD31	FITC-conjugated	BD	560984
	CD34	APC-conjugated	BD	560940
	CD44	PE-conjugated	BD	550989
	CD45	APC-conjugated	BD	340943
	CD73	PE-conjugated	BD	550257
	CD90	APC-conjugated	BD	561971
	CD105	PE-conjugated	BD	560839
	CD146	PE-conjugated	BD	550315
	SSEA4	PE-conjugated	BD	560128
	STRO-1	FITC-conjugated	Biolegend	340105
	HLA	Rabbit monoclonal	Abcam	52922
	CD68	Mouse monoclonal	AbD Serotec	MCA341R
	TGF-ßI	Rabbit monoclonal	Abcam	170874
	SOD-1	Rabbit monoclonal	Abcam	EP1727Y
				
	**Medium and Serum**		**Source**	**Catalog No.**
**Culture Mediums**	KSFM		Gibco	17005042
	DMEM		Gibco	41966-052
**Serums**	Fetal bovine serum		Gibco	26140079

**Table 2:** Pathohistological changes in kidney of USC-treated rats, compared to those in controls 12 weeks after cell therapy.

Pathological changes	PBS-treated Group*	USC-treated Group*	Age-matched Control*
	n=9	n=9	n=10
Ratio of normal nephrons (%) (Fig. 6)	38% (4.0±2.0)	60% (6.33±2.1)	100% (10.6±2.2)
Ratio of abnormal nephrons (%) (Fig. 6)	62% (6.6±2.0)	40% (4.27±2.1)	0% (0.0±0.0)
Ratio of collagen deposition (%) ( Fig. 7)	322% (16.2±4.7)	152% (7.6±3.3)	100% (5.0±2.0)
Ratio of inflammatory (%) (Fig. 8)	435% (8.7±2.5)	235% (4.7±2.1)	100% (2.0±1.1)
Increased interstitial fibrosis (cortex vs. medulla) (%) (Fig. 9)	158% (1.22±0.1) vs. 182% (1.32±0.08)	123% (0.95±0.09) vs. 128%(0.99±0.06)	100% (0.77±0.05) vs. 100%(0.72±0.06)

**Abbreviation**s: PBS- phosphate buffered saline; USC- urine-derived stem cells. **Notes**: Glomerular sclerosis, dilated or atrophic renal tubule structure; Inflammatory response - measured by monocyte infiltration. One-way ANOVA statistical analysis was formed among three groups, based on the average relative intensity of ten fields per sample, each taken at a 200x magnification for immunostaining. Percentages were relative to AMC. All values are reported as mean ± SEM. *All differences listed in this table were statistically significant between the PBS, USC-treated groups, and AMC, *p<0.05 or p<0.01*.

## Abbreviations

AMC: age-matched control
CKD: chronic kidney disease
ESRD: end-stage renal disease
H&E: hematoxylin and eosin
HLA: human leukocyte antigen
HPF: high power field
FITC: Fluorescein isothiocyanate
GFR: Glomerulus filtration rate
iPSC: induced pluripotent stem cells
PBS: phosphate buffered saline
PE: phycoerythrin
SSEA-4: stage- specific embryonic antigen-4
SPSS: Statistical Package for the Social Sciences
USC: Urine-derived stem cells
