# Supplementary material for: Reno-protection of Urine-derived Stem Cells in A Chronic Kidney Disease Rat Model Induced by Renal Ischemia and Nephrotoxicity
Source: Int J Biol Sci. 2020 Jan 1;16(3):435–46. doi: 10.7150/ijbs.37550 (PMC6990904; doi:10.7150/ijbs.37550)
Supplement: Supplementary file 1 — Supplementary figures and tables. [file ijbsv16p0435s1.pdf]

A

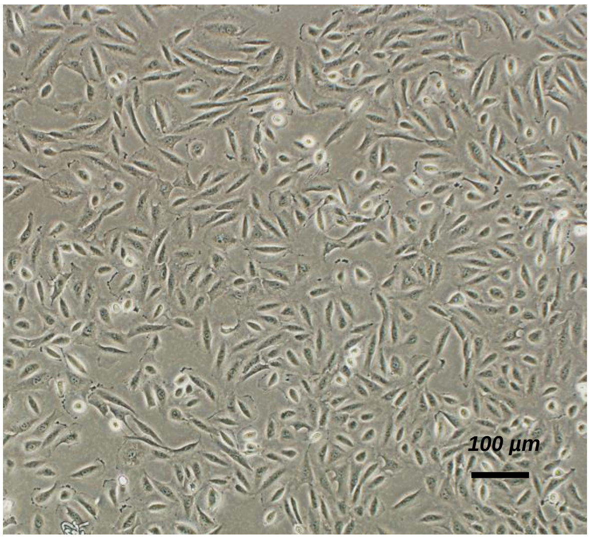

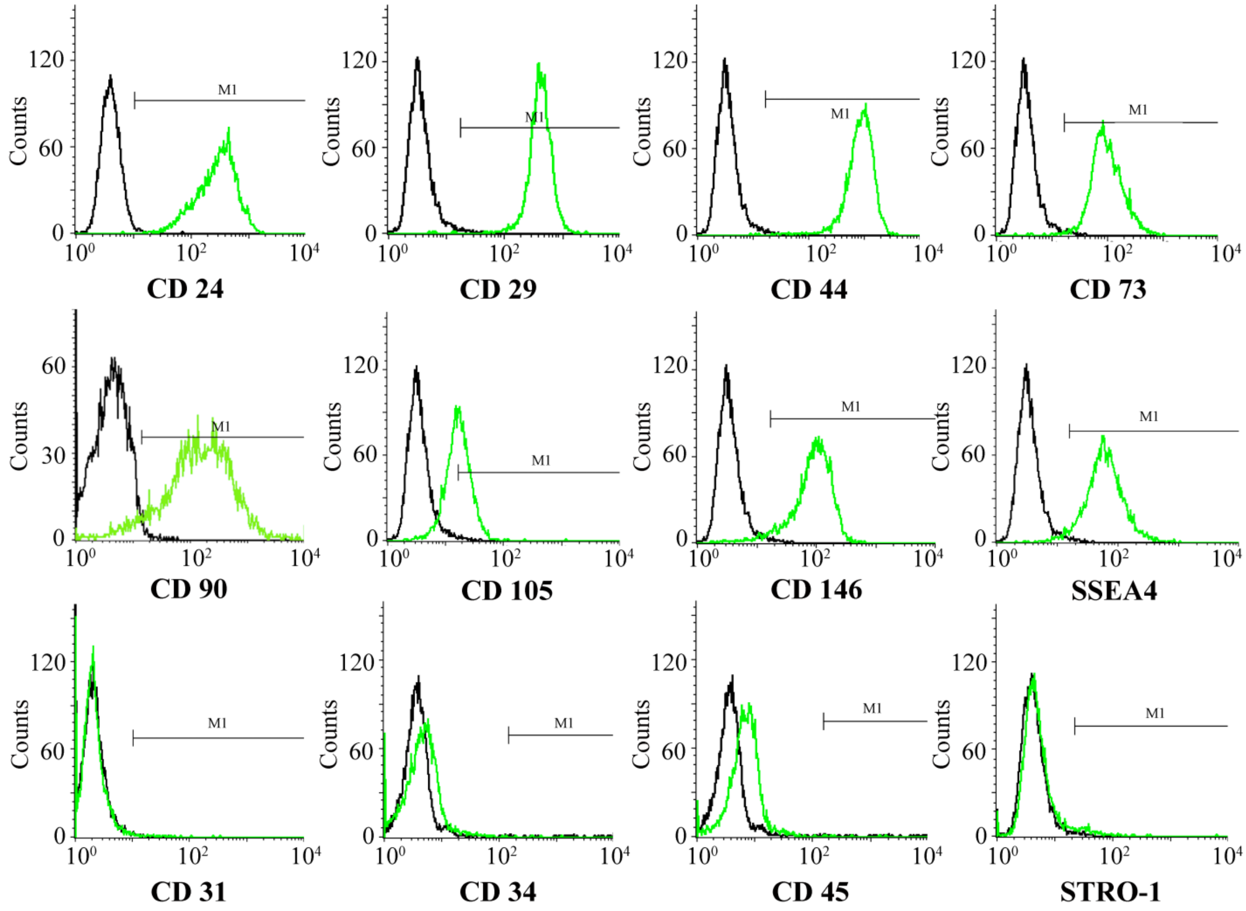

B

Supplement Figure 1. (**A**) Human USC all displayed ''rice grain''-like morphology in the early passage (*p2*). (**B**) USC expressed markers of glomerular parietal epithelial cells (CD24, CD29 and CD44), and also classical cell surface markers of MSC (CD73, CD90, CD105, CD146 and SSEA-4) and negative expression of hematopoietic markers (CD 31, CD34 and CD45).
